# Oxacillin sensitization of methicillin-resistant *Staphylococcus aureus* and methicillin-resistant *Staphylococcus pseudintermedius* by antisense peptide nucleic acids in vitro

**DOI:** 10.1186/s12866-015-0599-x

**Published:** 2015-11-11

**Authors:** Shan Goh, Anette Loeffler, David H. Lloyd, Sean P. Nair, Liam Good

**Affiliations:** Pathology and Pathogen Biology, Royal Veterinary College, Hawkshead Lane, Hatfield, Hertfordshire UK; Clinical Sciences and Services, Royal Veterinary College, Hawkshead Lane, Hatfield, Hertfordshire UK; Department of Microbial Diseases, UCL Eastman Dental Institute, University College London, London, UK; Pathology and Pathogen Biology, Royal Veterinary College, Royal College Street, London, UK

**Keywords:** Antisense, *mecA*, *ftsZ*, MRSA, Methicillin-resistant *Staphylococcus pseudintermedius*

## Abstract

**Background:**

Antibiotic resistance genes can be targeted by antisense agents, which can reduce their expression and thus restore cellular susceptibility to existing antibiotics. Antisense inhibitors can be gene and pathogen specific, or designed to inhibit a group of bacteria having conserved sequences within resistance genes. Here, we aimed to develop antisense peptide nucleic acids (PNAs) that could be used to effectively restore susceptibility to β-lactams in methicillin-resistant *Staphylococcus aureus* (MRSA) and methicillin-resistant *Staphylococcus pseudintermedius* (MRSP).

**Results:**

Antisense PNAs specific for conserved regions of the mobilisable gene *mecA*, and the growth essential gene, *ftsZ*, were designed. Clinical MRSA and MRSP strains of high oxacillin resistance were treated with PNAs and assayed for reduction in colony forming units on oxacillin plates, reduction in target gene mRNA levels, and cell size. Anti-*mecA* PNA at 7.5 and 2.5 μM reduced *mecA* mRNA in MRSA and MRSP (*p* < 0.05). At these PNA concentrations, 66 % of MRSA and 92 % of MRSP cells were killed by oxacillin (*p* < 0.01). Anti-*ftsZ* PNA at 7.5 and 2.5 μM reduced *ftsZ* mRNA in MRSA and MRSP, respectively (*p* ≤ 0.05). At these PNA concentrations, 86 % of MRSA cells and 95 % of MRSP cells were killed by oxacillin (*p* < 0.05). Anti-*ftsZ* PNAs resulted in swelling of bacterial cells. Scrambled PNA controls did not affect MRSA but sensitized MRSP moderately to oxacillin without affecting mRNA levels.

**Conclusions:**

The antisense PNAs effects observed provide in vitro proof of concept that this approach can be used to reverse β-lactam resistance in staphylococci. Further studies are warranted as clinical treatment alternatives are needed.

**Electronic supplementary material:**

The online version of this article (doi:10.1186/s12866-015-0599-x) contains supplementary material, which is available to authorized users.

## Background

Antimicrobial resistant bacteria threaten the health of humans and animals by limiting treatment options during infection [1]. Resistance can arise through spontaneous mutation or through the acquisition of resistance genes by horizontal transfer [2]. Given limited success in the development of new antimicrobial drugs, there is great interest in targeting resistance genes to re-establish susceptibility to existing antibiotics [3, 4]. This strategy is attractive because it would reduce the need to develop new classes of antimicrobials, but it requires the development of clinically effective and safe inhibitors of resistance. Several laboratories have pursued the idea of reducing expression of resistance genes so that the amount of protein conferring resistance is reduced to a level that re-sensitizes the bacterium [5]. If successful, this approach could provide a generic strategy applicable to a wide range of resistance genes that cause clinical problems.

Staphylococcal infections are amongst the most common presentations in human and veterinary medicine that lead to prescription of antimicrobial drugs [6–8]. The predominant staphylococcal species involved with disease would typically be susceptible to most classes of antimicrobial agents, e.g. *S. aureus* in humans and *S. pseudintermedius* in pets. However their methicillin-resistant counterparts, MRSA and MRSP, tend to be resistant to most clinically relevant antimicrobial drugs and both species have zoonotic potential [9, 10]. In MRSA and MRSP, resistance to methicillin and other β-lactams is conferred by mutated versions of the penicillin-binding protein (PBP2a). PBP2a is located within the bacterial cell wall and has low affinity to all β-lactam-antimicrobials [11], rendering them ineffective [12]. PBP2a is encoded by *mecA*, which is conserved amongst different staphylococcal species and located on a staphylococcal cassette chromosome (SCC). Hence it may be possible to develop a common strategy for inhibiting *mec*A expression in these pathogens and re-sensitize staphylococci.

PNAs are a type of synthetic DNA mimic that bind strongly to single-stranded nucleic acids, and are highly resistant to nucleases and proteases. They have been studied as antimicrobials through silencing of bacterial growth essential genes [5, 13, 14]; however growth inhibitory doses in MRSA were relatively high [14], risking non-specific effects. PNA has not been tested for reversing antibiotic resistance in staphylococci, but reversion by antisense phosphorothioate oligodeoxynucleotides (PS-ODN) and protein inhibitors has been reported [15, 16]. The use of antisense agents on MRSP has not been reported. The next step is to develop more potent inhibitors that can be considered for clinical development; e.g. topical medications to reverse resistance to beta-lactam drugs that are given systemically or locally. Although PNA synthesis is currently expensive, the relative risk and cost of finding and developing new classes of antibiotics could be higher [17]. If used clinically, the cost of large scale PNA synthesis is likely to reduce with advancing technology and methods. This was the case for penicillin, where the cost of a dose reduced from US$20 in 1943 (equivalent to US$287 in 2014) to US$0.25 in 2014 [18].

In this study we tested whether or not antisense PNA against two gene targets, *mecA* and *ftsZ*, could sensitize MRSA and MRSP to oxacillin. The anti-*mecA* PNA resulted in sensitization to oxacillin and *mecA* mRNA reduction in both MRSA and MRSP. The anti-*ftsZ* PNA resulted in cell swelling, growth inhibition, *ftsZ* mRNA reduction, and sensitization to oxacillin at lower concentrations than previously reported.

## Methods

### Bacterial growth

The MRSA strain NCTC 13142 from Public Health England Culture Collections is a UK epidemic strain type EMRSA-15, SCC*mec* Type IV, and the MRSP strain HH-1 was isolated from a canine skin infection and is ST71, SCC*mec*II-III [19]. Bacteria were cultured on Columbia base agar (Oxoid, Basingstoke, UK) supplemented with 5 % horse blood (Oxoid) and 6 μg/ml oxacillin (Sigma Aldrich, UK) at 35 °C for 20 h. Overnight broth cultures were prepared from single bacterial colonies inoculated in 3 ml Mueller Hinton broth (MHB, Oxoid) supplemented with 6 μg/ml oxacillin and incubated at 35 °C for 20 h at 180 rpm on a rotary shaker.

### Antibiotic susceptibility assays

The MIC of oxacillin was determined by broth microdilution according to CLSI guidelines [20]. Briefly, overnight broth cultures of MRSA and MRSP were standardized to OD_625_ = 0.08–0.09 (equivalent to 10^8^ CFU/ml) in MHB and further diluted 100-fold to obtain 10^6^ CFU/ml. The bacterial suspension was then inoculated at 50 μl per well to a final concentration of 5 × 10^5^ CFU/ml. Two-fold serial dilutions of oxacillin (2–512 μg/ml) were added to bacterial cultures at 50 μl per well to a final volume of 100 μl per well, and 96 well plates were incubated at 35 °C for 24 h. MICs were visually scored.

### Antisense PNA design

To design antisense PNAs complementary to *mecA* or *ftsZ* mRNAs in both MRSA and MRSP, the −20 to +20 region of the start codon that included the Shine Dalgarno (SD) and 5′ end sequences of *mecA* or *ftsZ* from several isolates of MRSA and MRSP were aligned using ClustalW2. Consensus sequences of conserved regions were analysed for specificity in the MRSA N315 genome in the GenoList database [21]. Although this database has limited genomes, we used it for sequence alignments to relevant SD regions of a genome in an antisense orientation, with specified numbers of mismatch bases.

### Genomic DNA extraction and PCR

Genomic DNA (gDNA) was extracted from 1 ml of overnight cultures using the Gentra Puregene Yeast/Bacteria kit (Qiagen). PCR was carried out on gDNA to determine the sequence of the *mecA* PNA target region (i.e. SD region and 5′ end of *mecA*) with primers mecA-99F and mecA-528R for both NCTC 13142 and HH-1 (Additional file 1: Table S1). To determine the sequence of the *ftsZ* PNA target regions (i.e. SD region and 5′ end of *ftsZ*), primers E15ftsZ_F and E15ftsZ_R specific for NCTC 13142, and ED99ftsZ_F and EDftsZ_R specific for HH-1 were used (Additional file 1: Table S1). PCR was carried out with Crimson Taq DNA polymerase (New England Biolabs) with cycling conditions of 95 °C for 5 min, 35 cycles of 95 °C for 30 s, 52 °C (*mecA* primers) or 61 °C (*ftsZ* primers) for 30 s, 68 °C for 1 min, and a final extension of 68 °C for 5 min.

### Oxacillin sensitization assays

Overnight broth cultures were adjusted to 5 × 10^5^ CFU/ml per well of a 96 well plate as described above and used either in a broth growth assay or a viable count assay. The broth growth assay was used as an initial screen of PNA efficacy because it was simple to carry out. In the broth assay, $$ {\scriptscriptstyle \raisebox{1ex}{$1$}\!\left/ \!\raisebox{-1ex}{$8$}\right.} $$, ¼, ½, 1 × MIC of oxacillin was used in the bacterial cultures (i.e. 64, 128, 256, 512 μg/ml oxacillin for NCTC 13142 or 128, 256, 512, 1024 μg/ml oxacillin for HH-1). Anti-*mecA* PNAs were added at either 2.5 or 5 μM. After incubation at 35 °C for 24 h, the MIC of oxacillin in combination with the PNAs in broth cultures were scored visually as recommended by CLSI guidelines [20].

The viable count assay is more sensitive than the broth assay and was used to assess the effects of PNAs that performed well in the initial screen described above. In the viable count assay, 5 × 10^5^ CFU/ml per well of culture was treated either with 2.5, 5 and 7.5 μM of PNA for NCTC 13142, or 1, 2.5 and 5 μM of PNA for HH-1 in MHB at 35 °C for 6 h. Cultures were serially diluted in PBS and 100 μl of 10^−3^, 10^−4^, 10^−5^, 10^−6^ dilutions were spread-plated onto MH agar supplemented with 16 μg/ml oxacillin, which was determined to be the highest selective but not growth inhibitory concentration for this assay. Agar plates were incubated at 35 °C for 24 h, and dilutions resulting in 30–300 colonies per plate were used to determine the viable count. Experimental repeats were carried out on separate days, and reductions in CFU of treated cultures were normalized to CFU of the untreated control culture on the day. Statistical analysis of CFU reductions was carried out with the paired Student’s *t*-Test in Microsoft Excel.

### Growth inhibition assays

Anti-*ftsZ* PNAs were tested for effects on growth. Overnight MHB cultures were adjusted to 5 × 10^5^ CFU/ml per well of a 96 well plate in MHB supplemented with 6 μg/ml oxacillin as described above. Anti-*ftsZ* PNA was added to final concentrations of 0, 1, 2.5, 5, 7.5 μM and incubated in a microplate reader (Spectramax 340PC, Molecular Devices) at 35 °C for 6 h. The optical density of the cultures was monitored at 550 nm with readings every 5 min after shaking for 5 s.

### Light microscopy

Bacterial cultures were treated with anti-*ftsZ* PNA Z46 in broth as described in the viable count assay. After 6 h at 35 °C, cultures were pelleted, washed once in PBS, and re-suspended in 1/10 of original volume of the culture in PBS. Ten microliter aliquots were heat-fixed, Gram stained, and viewed under a light microscope (Olympus BX60) at 1000 × magnification. Images were captured using the Image-Pro Plus 5.0 (MediaCybernetics), and counting of purple and pink cells were carried out using ImageJ 1.44o (National Institute of Health, USA).

### RNA extraction, cDNA synthesis and qPCR

Growth of cultures in a 96 well plate was monitored by reading the optical density at 550 nm every 5 min, after shaking for 5 s in a microplate reader (Spectramax 340PC, Molecular Diagnostics). Cultures were harvested when the untreated control culture increased in OD_550_ ≈ 0.1. Cultures with the same PNA treatment but in different wells across the 96 well plate were pooled for total RNA extraction and DNase I treatment (Ribopure™ Bacteria kit, Life Technologies). RNA was checked for gDNA contamination by qPCR using *gyrA* primers (Additional file 1: Table S1) and SsoFast™ EvaGreen® Supermix (Bio-Rad) before cDNA synthesis using 50 ng RNA and iScript™ Reverse Transcription Supermix (Bio-Rad). Samples of cDNA were diluted 10-fold and 4 μl used in each qPCR reaction. Non-template controls were included for each primer pair in each run, and technical duplicates for each sample were included in each run.

### Validation of primers for qPCR

Primer pairs for qPCR were designed by Primer3 with a Tm of 60 °C and product sizes of 100–200 bp (Additional file 1: Table S1). Gene sequences were taken from genome assemblies of *S. aureus* HO 5096 0412 (GenBank NC_017763.1), an EMRSA-15 strain, and *S. pseudintermedius* ED99 (GenBank NC_017568.1), a methicillin sensitive *S. pseudintermedius* strain, as annotated genome sequences of MRSP strains were not publicly accessible at the time of assay design. Similarities of these gene sequences were later confirmed to match MRSP E140 (Genbank accession NZ_ANOI01000001) sequences. Primer concentrations (300–500 nM) were optimized for use in SsoFast™ EvaGreen® Supermix in a CFX96 Real-time PCR Detection System with CFX Manager v2 (Bio-Rad), with 5 ng cDNA per reaction. Primer efficiencies were determined using 10-fold serial dilutions of cDNA (10 pg-1 μg). Melt-curve analyses were carried out for all reactions.

### Validation of reference genes and analysis of mRNA levels by RT-qPCR

Total RNA and cDNA were prepared as described above. Raw Cq values were analysed in qbase + (Biogazelle, Belgium) with user-defined primer efficiencies (Additional file 1: Table S1) and expression stabilities (M value) of each candidate reference gene *fabD*, *gyrA*, *proC*, *pta*, *pyk*, *rho*, *sodA*, and *tpiA* were calculated as the average pairwise variation for each gene with all other tested genes [22]. Low M values indicate stable expression. Stepwise exclusion of the gene with the highest M value allowed ranking of the tested genes according to their expression stability, and from this the optimal number of reference genes was calculated [22]. To measure the expression levels of genes conditionally silenced by antisense PNA, Cq values were normalized against the two most stably expressed references genes with user-defined PCR efficiencies (Additional file 1: Table S1) in qbase + (Biogazelle). Relative mRNA levels were then calculated as induced normalized Cq/uninduced normalized Cq, and analysed with paired Student’s *t*-Test in Microsoft Excel.

## Results

### Anti-*mecA* PNA sensitization of MRSA and MRSP to oxacillin

Alignment of the *mecA* start codon region of selected *S. aureus* and *S. pseudintermedius* strains showed 100 % alignment in all except the USA300 and livestock-associated MRSA ST398 (Additional file 2: Figure S1). As we were interested in the major UK epidemic strain NCTC 13142 (EMRSA-15, ST22, SCC*mec*IV) [23, 24], and the first genetically well-characterized clinical MRSP strain, HH-1 (ST71, SCC*mec*II-III) [19], we based our antisense designs on sequence alignments on these strains. Sequences of the PNA target regions in NCTC 13142 and HH-1 were additionally determined by PCR and sequencing to confirm that the antisense design was appropriate. Four anti-*mecA* and one scrambled PNA were designed and conjugated to the cell-penetrating peptide (CPP) KFFKFFKFFK (Table 1). Initial screening at low and sub-optimal concentrations of the four anti-*mecA* PNAs showed that A73 was the most effective and sensitized NCTC 13142 and HH-1 broth cultures to oxacillin by 4–8 fold at 2.5–5 μM (Additional file 1: Table S2). More sensitive viable count assays were then carried out to further examine the activity of A73.

**Table 1:** Antisense PNAs used in this study

PNA	5′ − > 3′ sequence	Target gene (target region around ATG)
A55	KFFKFFKFFK-eg1-ttcatcaata-CONH2	*mecA* (−5 to +5)
A73	KFFKFFKFFK-O-catcaatatc-CONH2	*mecA* (−7 to +3)
A82	KFFKFFKFFK-eg1-atcaatatcc-CONH2	*mecA* (−8 to +2)
A101	KFFKFFKFFK-O-caatatcctc-CONH2	*mecA* (−10 to −1)
scrA	KFFKFFKFFK-O-cactataatc-CONH2	N.A.
A73-EJH	MINWKLRLKNK-O-catcaatatc-CONH2	*mecA* (−7 to +3)
scrA-EJH	MINWKLRLKNK-O-cactataatc-CONH2	N.A.
A73-Tat	YGRKKRRQRRR-O-catcaatatc-CONH2	*mecA* (−7 to +3)
scrA-Tat	YGRKKRRQRRR-O-cactataatc-CONH2	N.A.
Z19	KFFKFFKFFK-eg1-TTCTAACATT-NH2	*ftsZ* (−1 to +9)
Z46	KFFKFFKFFK-eg1-TAACATTTAA-NH2	*ftsZ* (−4 to +6)
scrZ	KFFKFFKFFK-eg1-AATTACTATA-NH2	N.A.

The most effective antisense PNA (A73) and a scrambled sequence (scrA) were conjugated to two other CPPs to test for their ability to improve delivery: EJH-L1 (MINWKLRLKNK) and HIV 1-Tat (YGRKKRRQRRR) (Table 1). The (RXR)_4_XB peptide was not included because it could inhibit growth independently of the antisense PNA [14]. The EJH-L1 peptide was derived from a *Streptococcus pneumoniae* phage holin protein, shown to make lesions on lipid vesicles and cause cell death in *S. pneumoniae* and *E. coli* [25]. The HIV-1 Tat peptide improved antisense PNA effects compared to (KFF)_3_K in *Streptococcus pyogenes* [26] (Table 1). However, we observed that A73-EJH and A73-Tat PNAs sensitized NCTC 13142 or HH-1 to oxacillin only by 2-fold, while scrA-EJH and scrA-Tat did not have an effect in broth microdilution assays (data not shown). Since this was not an improvement over the sensitization observed using the (KFF)_3_K peptide as carrier, subsequent experiments were carried out using A73.

**Fig. 1:**
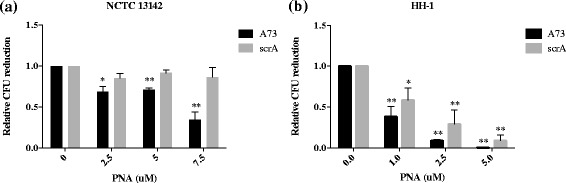
Sensitization of MRSA and MRSP to oxacillin by anti-*mecA* PNA. **a** Oxacillin resistant NCTC 13142 reduced in CFU with increasing concentrations of anti-*mecA* PNA, A73, while the scrambled PNA, scrA, did not have an effect. **b** Oxacillin-resistant HH-1 reduced in CFU with increasing concentrations of both A73 and scrA, with A73 being more effective. Relative CFU reduction was calculated as a ratio to an untreated control in each experiment. Values shown are mean ± SD, *n* = 3. *p < 0.05, **p < 0.01

An assay based on CFU of bacteria was used to quantify cell viability on oxacillin. The viability assay showed that treatment with A73 led to sensitization of NCTC 13142 and HH-1 to oxacillin (Fig. 1). NCTC 13142 cells were significantly sensitized to oxacillin at 2.5 μM (*p* = 0.015), 5 μM (*p* = 0.003) and 7.5 μM (*p* = 0.007) of A73 compared to the same concentrations of scrA (*p* = 0.062, *p* = 0.037, *p* = 0.122 respectively, Fig. 1). HH-1 cells were significantly sensitized to oxacillin at 1, 2.5 and 5 μM of A73 (*p* = 0.013, *p* = 1.15 × 10^−4^, *p* = 2.1 × 10^−5^ respectively, Fig. 1). ScrA also resulted in significant sensitization at the same concentrations (*p* = 0.04, *p* = 0.019, *p* = 0.002 respectively, Fig. 1).

### Anti-*mecA* PNA reduction of *mecA* mRNA

**Fig. 2:**
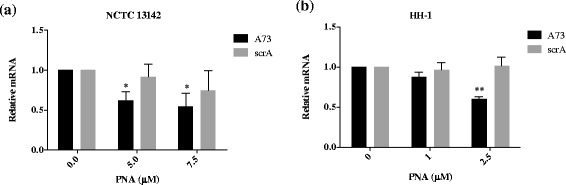
Reduction of *mecA* mRNA in MRSA and MRSP by anti-*mecA* PNA. Bacterial cultures were treated with indicated concentrations of anti-*mecA* PNA, A73, and scrambled PNA, scrA in the absence of oxacillin and grown to early log phase. RNA was extracted and *mecA* expression was quantified by qPCR, normalized to *pta* and *gyrA* for (**a**) NCTC 13142, and *proC* and *pyk* for (**b**) HH-1. Normalized mRNA values were calculated relative to the untreated control in each experiment, and plotted as mean ± SD of *n* = 3. *p < 0.05, **p < 0.01

To determine whether A73 specifically reduced *mecA* transcript levels, qPCR was carried out on PNA treated and untreated cultures. First it was important to determine the most appropriate MRSA and MRSP reference genes under these experimental conditions for normalization and relative quantitation of *mecA* mRNA levels. We tested the expression levels of seven candidate reference genes chosen from the literature [27, 28] on the following samples: NCTC 13142 untreated and treated with 5 and 7.5 μM of A73, scrA, Z46, scrZ, and HH-1 untreated and treated with 1 and 2.5 μM of A73, scrA, Z46 and scrZ. The two most stably expressed genes (i.e. having the lowest geNorm M-values, [29]) were *pta* and *gyrA* for NCTC 13142, and *proC* and *pyk* for HH-1 (Additional file 1: Table S3) [29]. These genes were used to normalize quantitative real-time PCR (qRT-PCR) data in NCTC 13142 and HH-1 samples, and showed that *mecA* mRNA was specifically reduced by A73 and not by scrambled PNA in both bacterial species (Fig. 2). Similar to the sensitization assay, a higher PNA dose was required for NCTC 13142 compared to HH-1: A73 at 5 μM reduced *mecA* transcripts in NCTC 13142 by an average of 38 % (*p* = 0.02), while 2.5 μM reduced *mecA* transcripts in HH-1 by an average of 40 % (*p* = 0.002). However, in contrast to the sensitization assay, the scrambled PNA did not have a significant effect in HH-1 (*p* = 0.42 for 2.5 μM, Fig. 2). For mRNA analysis, cultures were treated with A73 in the absence of oxacillin because the expression of *mecA* has been shown to increase in the presence of its protein inhibitors, such as oxacillin, particularly for isolates with functional regulatory genes, including HH-1 (ST71, SCCmecII-III) [30, 31]. To accurately quantify *mecA* silencing by PNAs, it was important to avoid possible oxacillin-mediated upregulation of *mecA*.

### Growth inhibition and sensitization of MRSA and MRSP to oxacillin by anti-*ftsZ* PNA

Inhibitors of the cell division protein FtsZ have been shown to sensitize MRSA to oxacillin [16]. In addition, FtsZ is of interest as an antimicrobial target as it is a cell division protein [32]. The *ftsZ* gene is essential for cell division and growth in *S. aureus*, and hypothesized to be essential in *S. pseudintermedius* based on bioinformatics analysis. Although the nucleotide sequence of *ftsZ* in *S. pseudintermedius* is different to that in *S. aureus*, the *ftsZ* SD region (i.e. −20 to +20 about start codon) was 95 % conserved across selected MRSA and *S. pseudintermedius* strains (Additional file 2: Figure S2). This provides an opportunity to design PNAs that can target *ftsZ* in both bacterial species. Inhibition of FtsZ protein leads to a defect in cell division, resulting in growth inhibition and swollen cell morphology [33], and we predicted that silencing of *ftsZ* mRNA would have similar effects.

**Fig. 3:**
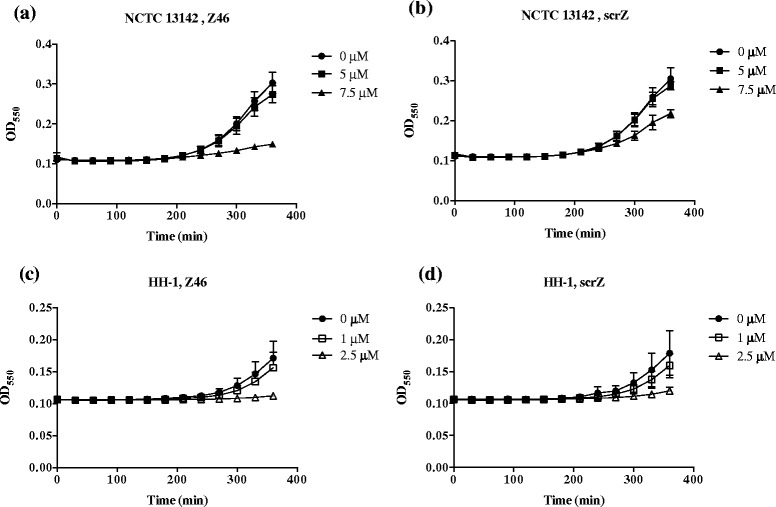
Phenotypic growth effect of anti-*ftsZ* PNA on MRSA and MRSP. Growth inhibition, an expected phenotypic effect of FtsZ depletion, was observed in NCTC 13142 at 7.5 μM of anti-*ftsZ* PNA, Z46 (**a**) but less so at the same concentration of the scrambled PNA, scrZ (**b**). Growth of HH-1 was equally inhibited at 2.5 μM of Z46 (**c**) and scrZ (**d**). Growth curves were plotted as mean OD550 ± SD, *n* = 3. *Filled circles* 0 μM, *open squares* 1 μM, *open triangles* 2.5 μM, *filled squares* 5 μM, *filled triangles* 7.5 μM

**Fig. 4:**
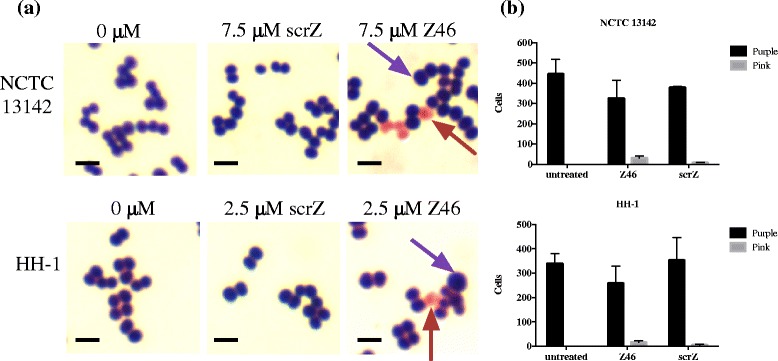
Phenotypic effect of anti-*ftsZ* PNA on cell morphology of MRSA and MRSP. **a** Swollen cells (*purple arrow*), an expected phenotypic effect of FtsZ were observed in Z46-treated cultures, while untreated and scrZ-treated cultures appeared normal. A larger number of Z46-treated cells stained pink (*red arrow*) compared to scrZ-treated cells; untreated cells did not stain pink. *Scale bar* = 1 μm. **b** Gram stained cells from three frames were counted with Image J and mean ± SD values are shown

Prior to testing anti-*ftsZ* PNAs for sensitization to oxacillin, we monitored effects on growth. Two anti-*ftsZ* and one scrambled (scrZ) PNA conjugated to the (KFF)_3_K peptide were tested on NCTC 13142 and HH-1 (Table 1). Z19 was ineffective against NCTC 13142 but Z46 inhibited NCTC 13142 growth at 7.5 μM and HH-1 growth at 2.5 μM (Fig. 3a & c). The scrambled PNA, scrZ, reduced the growth rate of NCTC 13142 at 7.5 μM but was not growth inhibitory (Fig. 3b). However, scrZ was growth inhibitory for HH-1 at 2.5 μM (Fig. 3d). Another phenotype associated with FtsZ depletion is cell swelling [32], hence Z46-treated cultures were Gram stained and examined microscopically. NCTC 13142 and HH-1 cells not only appeared swollen, but a greater proportion of cells also appeared lysed or stained pink at 7.5 and 2.5 μM, respectively, compared to untreated or scrZ-treated cells (Fig. 4a & b). The heterogeneous shape and staining of cells probably indicate different stages of cell death and cell wall disintegration. It has been shown that FtsZ recruits PBP2a in cell wall synthesis, and the depletion of FtsZ results in a loss of cell wall integrity [32].

**Fig. 5:**
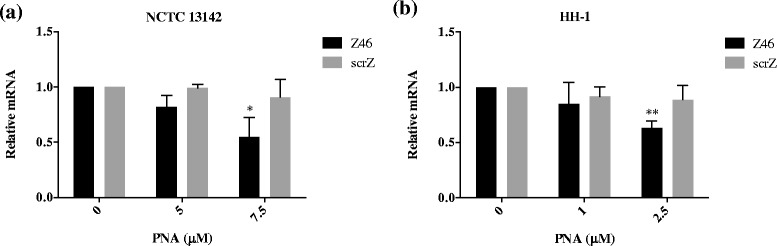
Reduction of *ftsZ* mRNA in MRSA and MRSP by anti-*ftsZ* PNA. Bacterial cultures were treated with indicated concentrations of anti-*ftsZ* PNA, Z46, and scrambled PNA, scrZ in the absence of oxacillin and grown to early log phase. RNA was extracted and *ftsZ* expression was quantified by qPCR, normalized to *pta* and *gyrA* for (**a**) NCTC 13142, and *proC* and *pyk* for (**b**) HH-1. Normalized mRNA values were calculated relative to the untreated control in each experiment, and plotted as mean ± SD of *n* = 3. *p < 0.05, **p < 0.01

**Fig. 6:**
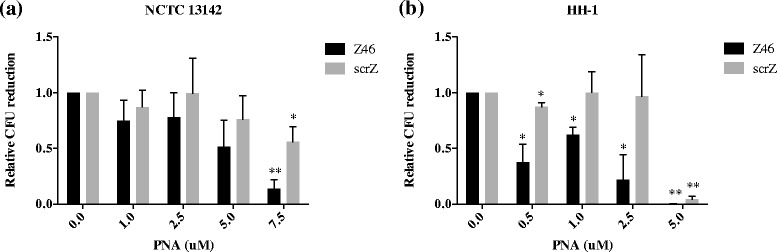
Sensitization of MRSA and MRSP to oxacillin by anti-*ftsZ* PNA. **a** CFU of NCTC 13142 was reduced by 87 % at 7.5 μM of anti-*ftsZ* PNA, Z46 compared to a reduction of 44 % by the scrambled PNA, scrZ. **b** CFU of HH-1 reduced significantly at all concentrations of Z46 compared to scrZ, except at 5 μM where Z46 and scrZ had similar effects. Relative CFU reduction was calculated as a ratio to an untreated control in each experiment. Values shown are mean ± SD, *n* = 3. *p < 0.05, **p < 0.01

Target gene product specificity of Z46 was shown through transcript analyses of Z46-treated cultures, which had reduced *ftsZ* mRNA levels in NCTC 13142 at 7.5 μM and HH-1 at 2.5 μM, compared to untreated or scrZ-treated cells (Fig. 5). Finally, we tested whether silencing of *ftsZ* had the same effect on cell sensitization to oxacillin as that reported for a FtsZ inhibitor [16]. Consistent with reversion of a resistance, we saw a reduction in CFU for all doses of silencer used, compared to an untreated control. Treatment with Z46 at 7.5 μM (*p* = 0.001) significantly sensitized NCTC 13142 to oxacillin compared to scrZ at 7.5 μM (*p* = 0.012, Fig. 6a). Treatment with Z46 at 0.5, 1 and 2.5 μM significantly sensitized HH-1 to oxacillin (*p* = 0.02, *p* = 0.01, *p* = 0.026, respectively) compared to scrZ at the same doses (*p* = 0.02, *p* = 0.98, *p* = 0.89, respectively, Fig. 6b).

## Discussion

We aimed to develop antisense PNAs that could provide potent and gene specific silencing of target genes within both MRSA and MRSP. As the CPP component of peptide-PNAs has been shown to improve potency, we tested two new peptide carriers in addition to the our standard KFF_3_K peptide: the HIV 1-Tat peptide and the EJH-L1 peptide, based on previously shown cell penetrating activities in related bacterial species. However, neither peptide resulted in an improvement in the silencing of *mec*A in NCTC 13142 and HH-1. It is known that teichoic acids in the peptidoglycan can be altered in D-alanine content to increase positive charge of cell walls, resulting in charge repulsion of and increased resistance against cationic antimicrobial peptides [34]. Indeed some MRSA strains possess thickened cell walls and increased D-alanine production [35]. It is possible that the NCTC 13142 strain tested in this study had similar cell wall alterations, reducing the activity of cationic CPPs conjugated to PNAs. A closer examination of cell wall properties of NCTC 13142, along with testing of CPPs of different structures may be required to identify CPPs that display comparable activity in Gram positive bacteria, as in Gram negative bacteria [36].

In the MRSP strain HH-1, PNAs were effective at much lower concentrations compared to NCTC 13142. This is presumably due to differences in cell wall structures, as passage across the cell wall is considered to be the main limiting factor for PNA efficacy. Another indication of cell wall differences is that scrambled PNAs affected cell viability of HH-1 without affecting transcript levels of *mecA* or *ftsZ* (i.e. non-specific toxic effect). A recent study showed the MIC of the (KFF)_3_K peptide against MRSP isolates was 2–8 μM [37], which support our observations of a non-specific toxic effect at 2.5 μM of peptide-conjugated PNA. There is limited information on the cell wall structure of MRSP, although genome comparisons of *S. pseudintermedius* to *S. aureus* identified unique cell wall proteins in *S. pseudintermedius* related to canine cell adherence [38]. However, differences in cell wall structure and components of *S. pseudintermedius*, MRSP and MRSA have not been examined. Hence it is difficult to speculate whether D-alanylation is contributing to the observed sensitivity to PNAs, as discussed for MRSA.

The A73 and Z46 PNAs specifically reduced target mRNA when corresponding scrambled controls did not (excluding general toxicity of 5 μM Z46 and scrZ on HH-1). Although mRNA reduction was low, the phenotype observed was consistent with gene requirement for resistance maintenance, indicating the important role(s) of *mecA* and/or *ftsZ* in resistance to oxacillin. This observation is similar to the effect of silencing *lacZ* and some essential genes in *Escherichia coli*, where a small reduction in mRNA results in a proportionally larger protein/phenotypic effect, due to translation repression or a high requirement for gene expression to maintain physiological functions [14, 39, 40]. In comparing the sensitization effects on epidemic MRSA and MRSP strains of high resistance (MIC ≥ 256 mg/L oxacillin) through silencing of two known targets (*mecA*, *ftsZ*), we found the mRNA levels of both gene targets in both species were reduced to similar levels with PNA treatment. Yet in NCTC 13142, silencing of *ftsZ* led to greater oxacillin sensitization, while in HH-1 silencing of *mecA* achieved greater sensitization. This may be due to the different ways in which *mecA* expression is regulated in NCTC 13142 and HH-1. NCTC 13142 harbours SCC*mec* IV, which contains a truncated *mecR1* and lacks *mecI* and *mecR2*. Hence *mecA* is constitutively expressed [31] and may be saturating anti-*mecA* PNA activity during cell division. HH-1 harbours SCC*mec* II-III, which contains intact regulatory genes *mecR1*, *mecI* and *mecR2* so that *mecA* expression is induced upon exposure to oxacillin [31]. As the sensitization assay involves exposing HH-1 to anti-*mecA* PNA before plating bacterial cultures onto oxacillin plates, it is less likely that the activity of anti-*mecA* PNA was saturated before cells divided. Alternatively, sensitization differences may be due to different growth kinetics for NCTC 13142 and HH-1. NCTC 13142 has a higher growth rate than HH-1 (Fig. 3), hence *ftsZ* silencing of NCTC 13142 could have a greater impact on cell division and cell wall synthesis leading to a greater sensitization effect.

Two previous studies examined the use of antisense PNAs in MRSA, targeting essential genes to achieve growth inhibition at 12.5–40 μM [14, 41]. This is the first study on the use of PNA for sensitizing MRSA and MRSP to oxacillin. Re-sensitization of MRSA to β-lactams was examined using an anti-*mecA* phosphorothioate oligonucleotide (PS-ODN) [15]. The data indicate that further improvements are needed, and studies on alternative technical approaches should increase the chances of success in resistance reversal strategies. Advantages of PNA over PS-ODN include higher binding affinity to RNA, greater convenience in formulation/usage as it does not require liposome encapsulation, and higher resistance to nucleases [42]. On the other hand, PS-ODNs are already used clinically for an anti-viral (Vitravene®), and an anti-cholesterol therapy (Kynamro®).

When developing topical medications for clinical use, bioavailability considerations are less relevant and PNAs have suitable properties because they are stable in biological fluids [43], effective when administered locally in murine models at concentrations similar to those used in this study [44], soluble in water at those concentrations, and have a long post-antibiotic effect [45]. The effective concentration of PNAs used in this study is the lowest reported for MRSA in vitro, at 7.5 μM, and the first reported for MRSP in vitro, at 2.5 μM. With molecular weights of 4211.5 and 4250.5 for A73 and Z46, respectively, 2.5–7.5 μM concentrations may be feasible for developing topical medication for clinical use. Hence these PNAs can be taken forward in further testing of toxicity and efficacy in vitro, ex vivo or in vivo in suitable animal models.

## Conclusions

Antisense PNAs targeting conserved regions in *mecA* and *ftsZ* of Staphylocci strains NCTC 13142 and HH-1 were able to re-sensitize cells to oxacillin, and inhibit the growth of cells at lower concentrations than previously reported. This study also shows for the first time that antisense PNAs are effective gene-silencers in a clinical MRSP isolate. However, a scrambled PNA affected HH-1 cell viability in a non-specific way, suggesting cell wall sensitivity to the CPP. It may be possible to use PNAs to develop pathogen selective antimicrobials for the topical treatment of skin infections. However, testing of additional antisense molecules against more clinical isolates of MRSA and MRSP is needed to evaluate the practicality of this approach.

## Additional files

Additional file 1: Table S1. Primers used in this study. **Table S2.** Oxacillin MIC (mg/L) of NCTC 13142 and HH-1 with anti-*mecA* PNA treatment (*n* = 2). **Table S3** Measure of candidate reference gene expression variability (M-value) by qPCR. (DOCX 119 kb) Additional file 2: Figure S1. Clustal Omega multiple sequence alignment of −20 to +20 of *mecA* of NCTC 13142 and HH-1 to selected epidemic MRSA and MRSP strains. HO_5096_0412 is an EMRSA-15 strain, MRSA252 is an EMRSA-16 strain, TW20 is a ST239 strain, N315 is a ST5 strain, USA300_FPR3757 is a ST8 strain, MRSAST398 is a livestock-associated strain. 06-3228, KM1381 and E140 are MRSP ST71 strains. Boxed sequences indicate target region of the A73 antisense PNA. **Figure S2.** Clustal Omega multiple sequence alignment of −20 to +20 of *ftsZ* of NCTC 13142 and HH-1 to selected epidemic MRSA and MRSP strains. HO_5096_0412 is an EMRSA-15 strain, MRSA252 is an EMRSA-16 strain, TW20 is a ST239 strain, N315 is a ST5 strain, USA300_FPR3757 is a ST8 strain, MRSAST398 is a livestock-associated strain. ED99 and HKU10-03 are both MSSP strains, E140 is an MRSP strain. Boxed sequences indicate target region of the Z46 antisense PNA. (PDF 127 kb)
