# Supplementary material for: Oxacillin sensitization of methicillin-resistant Staphylococcus aureus and methicillin-resistant Staphylococcus pseudintermedius by antisense peptide nucleic acids in vitro
Source: BMC Microbiol. 2015 Nov 11;15:262. doi: 10.1186/s12866-015-0599-x (PMC4642645; doi:10.1186/s12866-015-0599-x)
Supplement: Additional file 1: Table S1. — Primers used in this study. Table S2. Oxacillin MIC (mg/L) of NCTC 13142 and HH-1 with anti-mecA PNA treatment (n = 2). Table S3 Measure of candidate reference gene expression variability (M-value) by qPCR. (DOCX 119 kb) [file 12866_2015_599_MOESM1_ESM.docx]

**Table S1. Primers used in this study**

| Primer | Sequence | Target gene | Purpose |
| --- | --- | --- | --- |
| mecA-99F | cttctacacctccatatcacaa | *mecA* | Confirm sequence of PNA target region in  MRSA and MRSP |
| mecA528R | atgtatgctttggtctttctgc |  |  |
| E15ftsZ_F | gcggatttgattggtttaaacgt | *ftsZ* | Confirm sequence of PNA target region in  MRSA |
| E15ftsZ_R | ctcctgctcctaaaccacgt |  |  |
| ED99ftsZ_F | tgaacacggagaacctaaaca | *ftsZ* | Confirm sequence of PNA target region in  MRSP |
| ED99ftsZ_R | tccacgtgttaacttttcacca |  |  |
| Q/fabD_F1 | ttcggcattattagcagcgc | NCTC 13142 *fabD* | qPCR, 500nM F1/500nM R1, E=98.4% |
| Q/fabD_R1 | acgtcagctgcaactaaacttg |  |  |
| Q/ftsZ_F1 | tcaacacagacggtcaagct | NCTC 13142 *ftsZ* | qPCR, 500nM F1/400nM R1, E=103.8 |
| Q/ftsZ_R1 | cctgctcctaaaccacgtgt |  |  |
| Q/gyrA_F1 | attgcagagctcgttcgtga | NCTC 13142 *gyrA* | qPCR, 500nM F1/500nM R1, E=96.5% |
| Q/gyrA_R1 | ataacgacacgcacaccagt |  |  |
| Q/mecA_F1 | tccaggaatgcagaaagacca | *mecA* | qPCR, 500nM F1/500nM R1, E=102.7% |
| Q/mecA_R1 | tgctgttcctgtattggcca |  |  |
| Q/proC_F1 | agctggtacgaaacttggtct | NCTC 13142 *proC* | qPCR, 500nM F1/500nM R1, E=101.3% |
| Q/proC_R1 | gccatgctcaaatctgaacgt |  |  |
| Q/pta_F1 | aagcgactgaagaacaagcg | NCTC 13142 *pta* | qPCR, 500nM F1/500nM R1, E=99.7% |
| Q/pta_R1 | tgctgcaccactaactaaacca |  |  |
| Q/pyk_F1 | tggtgaaactgctgctggtt | NCTC 13142 *pyk* | qPCR, 500nM F1/500nM R1, E=111.5% |
| Q/pyk_R1 | ttttgtaatcttgggctgcttca |  |  |
| Q/rho_F1 | tttgttagttggcgagcgtc | NCTC 13142 *rho* | qPCR, 500nM F1/500nM R1, E=103.7% |
| Q/rho_R1 | ggtgttctggtggttcgtca |  |  |
| Q/sodA_F1 | tctgggagttactttcaccaaac | NCTC 13142 *sodA* | qPCR, 500nM F1/500nM R1, E=99.6% |
| Q/sodA_R1 | ccaacctgaaccaaagcgtg |  |  |
| Q/tpiA_F1 | acgatgttgtaggtgagcaagt | NCTC 13142 *tpiA* | qPCR, 500nM F1/500nM R1, E=102.7% |
| Q/tpiA_R1 | ccagttccgattgcccagat |  |  |
| Q/SP_fabD_F1 | cgattggtcacagtttaggcg | HH-1 *fabD* | qPCR, 300nM F1/400nM R1, E=93% |
| Q/SP_fabD_R1 | gccatcaattcgccacgttt |  |  |
| Q/SP_ftsZ_F1 | acaaggtcactctggcttcg | HH-1 *ftsZ* | qPCR, 400nM F1/400nM R1, E=95.8% |
| Q/SP_ftsZ_R1 | aagtttgaccagtgtgccca |  |  |
| Q/SP_gyrA_F1 | ctgcttcaacataccgtgcg | HH-1 *gyrA* | qPCR, 500nM F1/500nM R1, E=95.9% |
| Q/SP_gyrA_R1 | gatcatgcgtgcttgtcgtc |  |  |
| Q/SP_proC_F1 | agcgataacaggaagtggcc | HH-1 *proC* | qPCR, 500nM F1/500nM R1, E=102.4% |
| Q/SP_proC_R1 | tcgattcttccacttgcgatttt |  |  |
| Q/SP_pta_F1 | aaatccaaggcgacgcaaac | HH-1 *pta* | qPCR, no amplification |
| Q/SP_pta_R1 | ggacaggaccaatggcatca |  |  |
| Q/SP_pyk_F1 | ggacctgaaatccgtacgca | HH-1 *pyk* | qPCR, 500nM F1/500nM R1, E=93.5% |
| Q/SP_pyk_R1 | aacttcctgcatgctcacga |  |  |
| Q/SP_rho_F1 | agcgtctcgtcgaaattggt | HH-1 *rho* | qPCR, 500nM F1/500nM R1, E=97.6% |
| Q/SP_rho_R1 | aacgaggttatacgcacggg |  |  |
| Q/SP_sodA_F1 | ttaatcgcaaacttagacagcgt | HH-1 *sodA* | qPCR, 400nM F1/500nM R1, E=97.3% |
| Q/SP_sodA_R1 | agagtgatttaagtgaccgcca |  |  |
| Q/SP_tpiA_F1 | gaacgaaatgtgtgcagcgg | HH-1 *tpiA* | qPCR, 400nM F1/400nM R1, E=96.4% |
| Q/SP_tpiA_R1 | ctggctttacactaccaccgt |  |  |

**Table S2. Oxacillin MIC (mg/L) of NCTC 13142 and HH-1 with anti-*mecA* PNA treatment (n=2)**

| PNA | NCTC 13142 | | HH-1 | |
| --- | --- | --- | --- | --- |
|  | 0 μM | 5 μM | 0 μM | 2.5 μM |
| A55 | 256 | 256 | 512 | 512 |
| A73 | 512 | 128 | 512 | 64 |
| A82 | 512 | 128 | 512 | 256 |
| A101 | 512 | 512 | 512 | 256 |

**Table S3. Measure of candidate reference gene expression variability (M-value) by qPCR**

| Gene | Gene product | Pathway | M-value^a^ | |
| --- | --- | --- | --- | --- |
|  |  |  | MRSA | MRSP |
| *fabD* | Malonyl CoA-acyl carrier protein transacylase | Fatty acid biosynthesis | 0.22 | N.D.^b^ |
| *gyrA* | DNA gyrase subunit A | Replication | 0.16 | 0.11 |
| *proC* | Pyrroline-5-carboxylate reductase | Amino acid biosynthesis | 0.403 | 0.104 |
| *pta* | Phosphate acetyltransferase | Acetate metabolism | 0.163 | N.D.^c^ |
| *pyk* | Pyruvate kinase | Glycolysis | N.D.^b^ | 0.094 |
| *rho* | Transcription termination factor Rho | Transcription | 0.178 | 0.15 |
| *sodA* | Superoxide dismutase | Metabolic detoxification | 0.564 | 0.204 |
| *tpiA* | Triose phosphate isomerase | Gluconeogenesis | 0.342 | 0.178 |

^a^ Genorm M-value is the average M value of all remaining reference genes upon stepwise exclusion of the most unstable gene (highest M value). Genes with very stable expression have geNorm M-values ≤ 0.2 (1).

^b^ PCR efficiency of primer pair was not within range of other primer pairs, hence excluded in qPCR analysis

^c^ Primers specific for *pta* did not result in amplication and were excluded in qPCR analysis

1. **Hellemans J, Mortier G, De Paepe A, Speleman F, Vandesompele J.** 2007. qBase relative quantification framework and software for management and automated analysis of real-time quantitative PCR data. Genome Biol **8:**R19.
