# Supplementary material for: Oxacillin sensitization of methicillin-resistant Staphylococcus aureus and methicillin-resistant Staphylococcus pseudintermedius by antisense peptide nucleic acids in vitro
Source: BMC Microbiol. 2015 Nov 11;15:262. doi: 10.1186/s12866-015-0599-x (PMC4642645; doi:10.1186/s12866-015-0599-x)
Supplement: Additional file 2: Figure S1. — Clustal Omega multiple sequence alignment of −20 to +20 of mecA of NCTC 13142 and HH-1 to selected epidemic MRSA and MRSP strains. HO_5096_0412 is an EMRSA-15 strain, MRSA252 is an EMRSA-16 strain, TW20 is a ST239 strain, N315 is a ST5 strain, USA300_FPR3757 is a ST8 strain, MRSAST398 is a livestock-associated strain. 06-3228, KM1381 and E140 are MRSP ST71 strains. Boxed sequences indicate target region of the A73 antisense PNA. Figure S2. Clustal Omega multiple sequence alignment of −20 to +20 of ftsZ of NCTC 13142 and HH-1 to selected epidemic MRSA and MRSP strains. HO_5096_0412 is an EMRSA-15 strain, MRSA252 is an EMRSA-16 strain, TW20 is a ST239 strain, N315 is a ST5 strain, USA300_FPR3757 is a ST8 strain, MRSAST398 is a livestock-associated strain. ED99 and HKU10-03 are both MSSP strains, E140 is an MRSP strain. Boxed sequences indicate target region of the Z46 antisense PNA. (PDF 127 kb) [file 12866_2015_599_MOESM2_ESM.pdf]

```
                  -20      -10       1       10         20
MRSAST398         --ACTGTAAGGAGTGAGATGATATGAGA-ATAGAACGAGTAGA
NCTC13142         CTTATATAAGGAGGATATTGATGAAAAAGATAAAAATTGT---
HO_5096_0412      CTTATATAAGGAGGATATTGATGAAAAAGATAAAAATTGT---
MRSA252           CTTATATAAGGAGGATATTGATGAAAAAGATAAAAATTGT---
TW20              CTTATATAAGGAGGATATTGATGAAAAAGATAAAAATTGT---
N315              CTTATATAAGGAGGATATTGATGAAAAAGATAAAAATTGT---
HH-1              CTTATATAAGGAGGATATTGATGAAAAAGATAAAAATTGT---
06-3228           CTTATATAAGGAGGATATTGATGAAAAAGATAAAAATTGT---
KM1381            CTTATATAAGGAGGATATTGATGAAAAAGATAAAAATTGT---
E140              CTTATATAAGGAGGATATTGATGAAAAAGATAAAAATTGT---
USA300_FPR3757    CTTATATAAGGAGTATATTGATGAAAAAGATAAAAATTGT---
                      * *******     ****   * * *** **   **
```

Figure S1. Clustal Omega multiple sequence alignment of -20 to +20 of *mecA* of NCTC 13142 and HH-1 to selected epidemic MRSA and MRSP strains. HO_5096_0412 is an EMRSA-15 strain, MRSA252 is an EMRSA-16 strain, TW20 is a ST239 strain, N315 is a ST5 strain, USA300_FPR3757 is a ST8 strain, MRSAST398 is a livestock-associated strain. 06-3228, KM1381 and E140 are MRSP ST71 strains. Boxed sequences indicate target region of the A73 antisense PNA.

```
                    -20       -10       1       10        20
NCTC13142           AAAACTAGGAGGAAATTTAAATGTTAGAATTTGAACAAGG
HO_5096_0412        AAAACTAGGAGGAAATTTAAATGTTAGAATTTGAACAAGG
MRSA252             AAAACTAGGAGGAAATTTAAATGTTAGAATTTGAACAAGG
TW20                AAAACTAGGAGGAAATTTAAATGTTAGAATTTGAACAAGG
MRSAST398           AAAACTAGGAGGAAATTTAAATGTTAGAATTTGAACAAGG
USA300_FPR3757      AAAACTAGGAGGAAATTTAAATGTTAGAATTTGAACAAGG
N315                AAAACTAGGAGGAAATTTAAATGTTAGAATTTGAACAAGG
HH-1                AAGATTAGGAGGAAATTTAAATGTTAGAATTTGAACAAGG
ED99                AAGATTAGGAGGAAATTTAAATGTTAGAATTTGAACAAGG
HKU10-03            AAGATTAGGAGGAAATTTAAATGTTAGAATTTGAACAAGG
E140                AAGATTAGGAGGAAATTTAAATGTTAGAATTTGAACAAGG
                    ** * ***********************************
```

Figure S2. Clustal Omega multiple sequence alignment of -20 to +20 of *ftsZ* of NCTC 13142 and HH-1 to selected epidemic MRSA and MRSP strains. HO_5096_0412 is an EMRSA-15 strain, MRSA252 is an EMRSA-16 strain, TW20 is a ST239 strain, N315 is a ST5 strain, USA300_FPR3757 is a ST8 strain, MRSAST398 is a livestock-associated strain. ED99 and HKU10-03 are both MSSP strains, E140 is an MRSP strain. Boxed sequences indicate target region of the Z46 antisense PNA.
